# Dietary micronutrient intakes among women of reproductive age in Mumbai slums

**DOI:** 10.1038/s41430-019-0429-6

**Published:** 2019-05-31

**Authors:** Rebecca L. Nunn, Sarah H. Kehoe, Harsha Chopra, Sirazul A. Sahariah, Meera Gandhi, Chiara Di Gravio, Patsy J. Coakley, Vanessa A. Cox, Harshad Sane, Devi Shivshankaran, Ella Marley-Zagar, Barrie M. Margetts, Alan A. Jackson, Ramesh D. Potdar, Caroline H. D. Fall

**Affiliations:** 1 MRC Lifecourse Epidemiology Unit, University of Southampton, Southampton, UK; 2 Centre for the Study of Social Change, Mumbai, India; 3 Public Health Nutrition, Faculty of Medicine, University of Southampton, Southampton, UK; 4 National Institute for Health Research, Southampton Biomedical Research Centre, Southampton, UK

**Keywords:** Epidemiology, Risk factors

## Abstract

**Objectives:**

To (1) describe micronutrient intakes among women of reproductive age living in Mumbai slums; (2) assess the adequacy of these intakes compared with reference values; (3) identify important dietary sources of micronutrients.

**Subjects/methods:**

Participants were 6426 non-pregnant women aged 16–39 years, registered in a randomised controlled trial of a food-based intervention set in the Bandra, Khar and Andheri areas of Mumbai, India. Cross-sectional quantified food frequency questionnaire (FFQ) data were collected. Vitamin (*n* = 9) and mineral (*n* = 6) intakes were calculated and analysed in relation to dietary reference values (DRVs). Important dietary sources were identified for each micronutrient.

**Results:**

Median intakes of all micronutrients, except vitamin E, were below the FAO/WHO reference nutrient intake (RNI). Intakes of calcium, iron, vitamin A and folate were furthest from the RNI. For seven of the micronutrients, over half of the women had intakes below the lower reference nutrient intake (LRNI); this figure was over 75% for calcium and riboflavin. The majority of women (93%) had intakes below the EAR for 5 or more micronutrients, and 64% for 10 or more. Adolescents had lower intakes than women aged >19 years. Less than 1% of adult women and no adolescents met the EAR for all micronutrients. Animal source foods and micronutrient-rich fruit and vegetables were consumed infrequently.

**Conclusions:**

These women had low intakes of multiple micronutrients, increasing their risk of insufficiency. There is a need to determine the factors causing poor intakes, to direct interventions that improve diet quality and nutritional sufficiency.

## Introduction

Micronutrient deficiencies are a serious public health problem among women of reproductive age in low- and middle-income countries (LMICs) [1–4]. WHO states that malnutrition is directly or indirectly linked to major causes of death and disability worldwide [5–7]. Over 2 billion people (most of whom live in LMICs) are estimated to be deficient in one or more vitamins and minerals, particularly iron, zinc and vitamin A [2, 4, 8, 9]. In India, iron, vitamin A and vitamin B12 deficiencies are widespread in low-income populations, children and pregnant women [10–12]. Micronutrient requirements increase during pregnancy. This can exacerbate existing insufficiencies resulting in adverse maternal outcomes [9, 13]. Poor-quality diets before pregnancy may contribute to birth defects, intrauterine growth restriction [1, 14] and infant stunting [15].

The main direct cause of micronutrient deficiencies is insufficient intakes [16]. Low bioavailability is also a factor for some micronutrients. Intakes are low when access to micronutrient-rich foods and fortified foods is limited [1, 17], usually because they are expensive [18], locally unavailable or unacceptable for cultural or religious reasons [19]. Diets in India are often poor quality, carbohydrate rich [20–22] and lacking in micronutrient-rich foods [11]. ‘Animal source’ foods are rich in protein, iron and vitamin B12, yet in the state of Maharashtra, fewer than 2% of women reported daily consumption of meat/chicken or fish and 35% never ate them [12]. Vegetarian diets diverse enough to provide adequate micronutrients may not be affordable. Fruit and vegetable intakes in India are often below the recommended five portions per day [22–25].

There is a lack of data on micronutrient intakes of women of reproductive age, and important dietary sources, in India. Most research has focused on rural populations [11, 26, 27], with few studies in urban settings [10]. Investigations have mainly focused on pregnant women; few have studied preconceptional women of reproductive age or compared intakes of adults and adolescents against the respective recommended intakes. Studies typically focus on iodine, iron, vitamin A, vitamin B12, folate, and vitamin C [10], but few have investigated a wide range of micronutrients linked to dietary sources [26, 28].

The aim of this study is to provide a unique insight into food and micronutrient consumption of a large cohort of women of reproductive age living in Mumbai slums. Our objectives were to (1) describe micronutrient intakes amongst these women; (2) assess the adequacy of these intakes against references; (3) identify important dietary sources of micronutrients.

## Methods

Data were collected during registration for the Mumbai Maternal Nutrition Project (MMNP), a randomised controlled efficacy trial in India to investigate the effects of daily consumption of a food-based micronutrient-rich supplement on infant outcomes [29].

### Setting and participants

The trial took place from January 2006 to May 2012 in the Bandra, Khar, Santa Cruz and Andheri areas of the city of Mumbai, India. The setting was the slum areas covered by the health and social programmes of the non-governmental organisation the Centre for the Study of Social Change (CSSC). Women were eligible if aged <40 years, married, non-pregnant, not sterilised, planning to have more children and intending to deliver in Mumbai. Women were recruited at a range of locations close to their homes. Community meetings were held to obtain community consent and answer questions (full recruitment methods in Potdar et al. [29]).

### Demographic and anthropometric data

Data on age, education, religion, first language, occupation and socio-economic status were collected by interview. Educational attainment was recorded in seven groups ranging from illiterate to post-graduate. Socio-economic status was assessed using the Standard of Living Index from the Indian National Family Health Survey, which is based on housing type, utilities and household possessions [30]. Weight and height were measured using standardised techniques. Height was measured to the nearest 0.1 cm using a Harpenden portable stadiometer (CMS Instruments Ltd, London, UK) and weight to the nearest 10 g using digital weighing scales (SECA, Hanover, MD, USA).

### Dietary data

Dietary intakes were assessed using a 212-item food frequency questionnaire (FFQ) developed specifically for MMNP. The FFQ was administered in Hindi or Marathi by a nutritionist or trained project assistant at the time of registration. Women were asked how often they had consumed each food item in the previous 7 days and the usual portion size. Data were recorded as frequency codes (D1 = once a day, D2 = twice a day, W1 = once a week etc.). Portion size was determined using standardised local measurements demonstrated visually to participants using a range of different sized spoons, bowls and chapatti (Indian unleavened bread) templates. All weights used in subsequent calculations were related to the edible proportion of the food.

### Nutrient intake calculations

Intake frequencies and reported portion sizes were used to calculate weekly intake weights of each food item for each woman. Dietary intakes of energy, macronutrients and micronutrients were calculated using McCance and Widdowson’s ‘The Composition of Foods’ integrated dataset versions 5 and 6 [31]. This programme was chosen because composition data were available for all foods for the nutrients of interest. For foods prepared at home (*n* = 164), at least two household recipes were obtained from women living in the study area, nutrient content was calculated as described below and values were averaged. For each recipe, all ingredients were weighed in the project kitchen to the nearest gram using electronic scales. The food was then cooked and the cooked dish was weighed. The nutrient content of cooked food was calculated as follows:

$${\text{Nutrient}}\;{\text{content}}\,{\text{per}}\,{\text{gram}}\,{\text{of}}\;{\mathrm{cooked}}\;{\mathrm{food}} = \frac{{{\mathrm{Total}}\;{\mathrm{nutrient}}\;{\mathrm{content}}\;{\mathrm{of}}\;{\mathrm{raw}}\;{\mathrm{ingredients}}}}{{{\mathrm{Weight}}\;{\mathrm{of}}\;{\mathrm{cooked}}\;{\mathrm{food}\,{(\text{g})}}}}$$

For each nutrient, we used the recommended conversion factors in the database to allow for nutrient losses, due to the method of cooking. For raw or unprepared items such as fruit or milk (*n* = 30), and for shop-bought items such as biscuits or packaged drinks (*n* = 28), values were taken directly from the database of foods that were the same or as close as possible in nutritional content to the FFQ items. For foods containing beta-carotene, a ratio of 6:1 was used to calculate retinol equivalents.

### Dietary reference values

The dietary reference values (DRVs) used for analysis came from two sources. Reference nutrient intakes (RNIs) were taken from FAO/WHO 2011 [32], as these were the most up-to-date international reference values. The Indian Council of Medical Research recommendations were not available for all of the nutrients of interest. On inspection, the values were similar to the FAO/WHO values. Estimated average requirements (EARs) and lower reference nutrient intakes (LRNIs) from the 1990 UK COMA report [33] were used, as values were not available from FAO/WHO. LRNI values were available for all micronutrients, except for vitamin E. Where COMA EARs were not available, vitamin E [34] and selenium [35] values from the American Institute of Medicine (IOM) Food and Nutrition Board were used. Bioavailability was assumed to be 10% for iron and ‘low’ for zinc, in line with bioavailability evidence [36, 37] and previous studies with similar populations/dietary patterns [16]. EARs were predominantly used for analysis, as they demonstrate adequacy for 50% of a population. Using the LRNI may show serious deficiency risk, but loses some detail of the data distribution and may overestimate the percentage of adequate intakes and underestimate inadequate intakes.

**Fig. 1:**
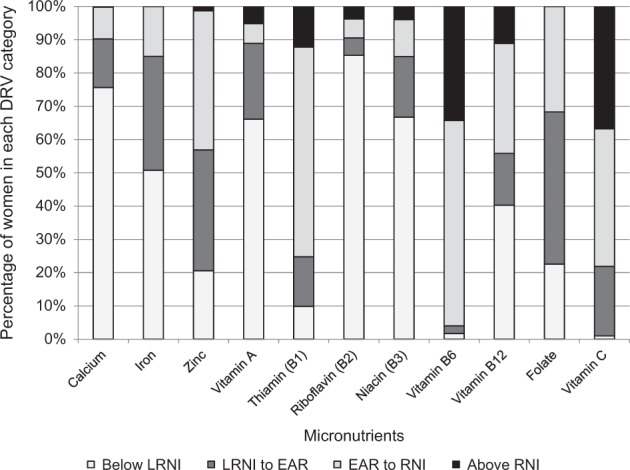
Percentage of women achieving micronutrient dietary reference value intakes. Vitamin E is not included as there is no LRNI. Selenium and magnesium are not included, as the LRNI, EAR and RNI do not occur in order, so they do not conform to the categories in the legend

### Data analysis

Data from women with total energy intakes outside the range of the mean ± 3SD (standard deviations) were excluded, as this suggests over- or under-reporting or an exceptional week. Descriptive data are presented as means and standard deviations for normally distributed variables, and median (IQR) for non-normally distributed variables. Ratios were calculated for the intake against the age-specific RNI of each micronutrient. As some DRVs are different for adolescents and adult women, daily micronutrient intakes, comparison of intakes with DRVs, multiple EARs not met and RNI ratios were analysed separately for adolescents (<19 years of age) and adult women (≥19 years of age). The chi-square test was used to investigate the differences between adults and adolescents in meeting the EAR (Fisher’s exact test if any cell had a count <5). Statistical significance was set at *p* = 0.05. Odds ratios, with 95% confidence intervals (CI), were calculated for the odds of the EAR being met by adults relative to adolescents. Statistical analyses were carried out using SPSS version 21.0 and STATA version 14.

FFQ foods were grouped into the following 10 categories for analysis: dishes made wholly or predominantly from (1) grains, roots or tubers; (2) pulses, legumes or nuts; (3) vegetables; (4) fruits; (5) fried snacks; (6) meat or meat products; (7) fish or shellfish; (8) eggs; (9) milk or other dairy products (not eggs); (10) sugar, sweets, condiments or beverages. Intakes of micronutrients were presented as the total intake from each food group, as a percentage of the total intake of that micronutrient.

## Results

### Descriptive characteristics

A total of 6513 women were eligible and participated in MMNP. We excluded 87 (1.4%) women from the analysis, because of implausible energy intakes. This left 6426 women with complete dietary data. Median age was 25 years and over three-quarters had completed at least secondary-level education (Table 1). The majority were of Hindu or Muslim faith and over half spoke Marathi as their first language. Recruitment took place throughout the year, though fewer women were recruited in the pre-monsoon season. The median (range; IQR) body mass index (BMI) was 20.0 (10.8–46.9; 17.9–22.9) kg/m^2^. Approximately half of the women were of normal BMI and a third were underweight.

**Table 1:** Descriptive characteristics of women studied

	Median	IQR
Age (years) (*n* = 6426)	25	22–28
BMI (kg/m^2^) (*n* = 6420)	20.0	17.9–22.9
	Mean	SD
Standard of Living Index score (*n* = 6077)	24.5	6.1
	*n*	%
*Age* (*n* = 6426)		
Aged 16–18 years	188	2.9
Aged ≥19 years	6238	97.1
*Education* (*n* = 6419)		
Postgraduate, graduate and higher secondary education	1130	17.6
Secondary education	4491	70.0
All other (including primary and illiterate)	797	12.4
*Religion* (*n* = 6420)		
Hindu	4525	70.5
Muslim	1620	25.2
Other	275	4.3
*First language* (*n* = 6416)		
Marathi	3314	51.7
Hindi	2408	37.5
Other	694	10.8
*Year of recruitment* (*n* = 6425)		
2006	1289	20.1
2007	1418	22.1
2008	1272	19.8
2009	1148	17.9
2010	1145	17.8
2011	153	2.4
*Season of recruitment* (*n* = 6425)		
Winter (January–February)	1467	22.8
Pre-monsoon (March–May)	1146	17.8
Monsoon (June–September)	2199	34.2
Post monsoon (October–December)	1613	25.1
*BMI* (*n* = 6420)		
Underweight (BMI <18.5 kg/m^2^)	2060	32.1
Normal range (BMI 18.5–25 kg/m^2^)	3446	53.6
Overweight (BMI >25 kg/m^2^)	737	11.5
Obese (BMI >30 kg/m^2^)	177	2.8

### Food group consumption

Dishes predominantly made of grains, roots or tubers were consumed frequently, as were dishes containing pulses, legumes or nuts (Table 2). These two food groups contributed the largest proportion of energy. Mean intake frequencies of fruits and vegetables were low in relation to Indian food-based recommendations [38]. Approximately 80% of women consumed both in the 7-day period, but 50% ate fruit no more than twice per week. Fried snacks were frequently consumed; at least 25% of women consumed them at least four times per week.

**Table 2:** The frequency of consumption of food groups and their contribution to total energy intake

Food group	Contribution to total energy (%)	Intake frequency per week Median (IQR)	Women consuming this food group in the last week (%)
1. Grains, roots or tubers	59.6	33 (28, 39)	99.9
2. Pulses, legumes or nuts	11.7	14 (8, 19)	99.6
3. Vegetables	1.9	8 (3, 11)	95.0
4. Fruits	2.0	2 (1, 5)	84.7
5. Fried snacks	3.4	2 (1, 4)	77.0
6. Meat or meat products	5.8	1 (0, 2)	58.3
7. Fish or shellfish	1.6	0 (0, 1)	46.4
8. Eggs	1.2	1 (0, 1)	51.4
9. Milk or other dairy products	7.3	14 (13, 17)	95.4
10. Sugar, sweets, condiments or beverages	5.4	3 (1, 7)	89.0

The mean consumption frequency of meat and meat products was low. The frequency of intakes of eggs, fish and shellfish dishes were low in relation to Indian food-based dietary guidelines [38] and their contribution to energy intakes was also low. Over 50% of women did not consume fish or shellfish and at least 25% did not consume eggs. In total, 75% of women consumed milk and milk products at least 13 times in the 7-day period, of which around 50% was tea or coffee with milk. In total, 50% of women consumed sugar, sweets, condiments and beverages three or more times.

### Micronutrient intakes and dietary reference values

Dietary intakes were low in relation to recommendations for most of the micronutrients studied, with the exception of vitamins B6, C and E (Table 3). Median intakes of all micronutrients, except vitamin E, were below the RNI. Intakes of calcium, iron, vitamin A and folate were furthest from the RNI, each with a median ratio below 0.4. For magnesium, 43.9% of all women had intakes below the LRNI, only 16.5% of adolescents met the EAR and 24.5% met the slightly lower RNI. For selenium, 66.1% of women were consuming intakes below the RNI [32], which is lower than the EAR [35] and LRNI, and 93.2% were consuming below the EAR. There was variation in the proportion achieving different levels of DRVs for each micronutrient (Fig. 1). The profiles of calcium and riboflavin were the poorest, with over 75% not meeting the LRNI. Over half were consuming below the LRNI of calcium, iron, selenium, vitamin A, riboflavin and niacin. Over 80% were consuming below the EAR for calcium, iron, selenium, vitamin A, riboflavin and niacin. Over 50% met the EAR for thiamine, vitamin B6 and vitamin C. Significantly fewer adolescents than adults were meeting the EAR for calcium, magnesium, zinc, thiamine, niacin, vitamin B6 and vitamin E (*p* < 0.05 for all). Odds ratios for the EAR being met (adults compared with adolescents) were as follows: calcium OR 10.4 (95% CI: 2.6, 41.8), magnesium 3.9 (2.6, 5.7), zinc 1.5 (1.1, 2.0), thiamine 2.0 (1.5, 2.7), niacin 2.4 (1.4, 4.3), vitamin B6 1.8 (1.0, 3.2) and vitamin E 1.4 (1.1, 1.9).

**Table 3:** Micronutrients—recommendations and summary intakes for adolescents aged 15–18 years (*n* = 188) and adult women (*n* = 6238)

Micronutrient (unit)	Age (years)	EAR (per day)	RNI (per day)	Intake (median, IQR)	Percentage of women with intakes		Ratio of intake to RNI (median, IQR)
					>EAR	>RNI	
Calcium (mg)	15–18	625	1300	280 (189, 386)	1.1	0	0.22 (0.15, 0.30)
	19–50	525	1000	293 (221, 398)	1.0	0.2	0.29 (0.22, 0.40)
Iron (mg)^a^	15–18	11.4	31	7.2 (5.3, 9.6)	12.2	0	0.23 (0.17, 0.31)
	19–50	11.4	29.4	7.9 (6.2, 10.1)	15.1	0	0.27 (0.21, 0.34)
Magnesium (mg)	15–18	250^b^	220^b^	171 (123, 220)	16.5^b^	24.5^b^	0.78 (0.56, 0.99)
	19–50	200	220	189 (148, 237)	43.3	32.2	0.86 (0.67, 1.08)
Selenium (μg)	15–18	45^c^	26	22.8 (16.9, 28.9)	3.2	31.4	0.88 (0.65, 1.11)
	19–50	45^c^	26	22.5 (17.8, 28.8)	3.8	33.8	0.87 (0.69, 1.11)
Zinc (mg)	15–18	5.5	14.4	4.80 (3.69, 6.14)	34.6	0	0.33 (0.26, 0.43)
	19–50	5.5	9.8	5.24 (4.20, 6.39)	43.4	1.4	0.58 (0.47, 0.71)
Vitamin A (μg RE)	15–18	400	600	198 (135, 296)	11.7	3.2	0.33 (0.23, 0.49)
	19–50	400	500	196 (131, 290)	11.1	5.2	0.39 (0.26, 0.58)
Thiamine (B1) (mg)^d^	15–18	0.63	1.0	0.69 (0.49, 0.88)	60.6	18.1	0.69 (0.49, 0.88)
	19–50	0.58	1.1	0.75 (0.58, 0.94)	75.7	12.1	0.68 (0.53, 0.85)
Riboflavin (B2) (mg)	15–18	0.9	1.0	0.48 (0.33, 0.64)	5.9	2.7	0.47 (0.33, 0.64)
	19–50	0.9	1.1	0.5 (0.37, 0.67)	9.6	3.8	0.45 (0.34, 0.61)
Niacin (B3) (mg NE)^e^	15–18	11.6	16	5.42 (3.73, 8.55)	6.9	1.6	0.34 (0.23, 0.54)
	19–50	10.7	14	6.99 (4.73, 9.28)	15.3	4.0	0.50 (0.34, 0.66)
Vitamin B6 (mg)^f^	15–18	0.6	1.2	1.09 (0.83, 1.33)	93.1	36.7	0.91 (0.69, 1.11)
	19–50	0.6	1.3	1.15 (0.93, 1.41)	96.1	34.2	0.89 (0.71, 1.08)
Vitamin B12 (µg)	15–18	1.25	2.4	1.09 (0.68, 1.54)	41.0	7.4	0.46 (0.28, 0.64)
	19–50	1.25	2.4	1.15 (0.73, 1.73)	44.2	11.2	0.48 (0.30, 0.72)
Folate (µg DFE)	15–18	150	400	119 (92, 155)	26.6	0	0.30 (0.23, 0.39)
	19–50	150	400	130 (103, 161)	31.9	0	0.32 (0.26, 0.40)
Vitamin C (mg)	15–18	25	40	36.3 (24.6, 57.2)	73.4	43.6	0.91 (0.62, 1.43)
	19–50	25	45	37.3 (26.4, 54.5)	78.3	36.6	0.83 (0.59, 1.21)
Vitamin E (mg α-TE)	15–18	12^c^	7.5	10.5 (7.79, 14.7)	40.4	79.3	1.39 (1.05, 1.96)
	19–50	12^c^	7.5	11.9 (8.98, 15.7)	49.3	86.0	1.59 (1.20, 2.09)
Energy (kcal)	15–18	2110 ^g^	–	1261 (996, 1590)	6.4	–	0.60 (0.47, 0.75)^h^
	19–50	1940^g^	–	1299 (1052, 1601)	9.7	–	0.67 (0.54, 0.83)^h^

^a^Iron bioavailability assumed at 10%

^b^For magnesium, the values for adolescent EAR are higher than the adolescent RNI value

^c^EARs from Food and Nutrition Board, Institute of Medicine and National Academies [34, 35], not COMA [33]

^d^Thiamine DRVs calculated using LRNI based upon 0.23 mg/1000 kcals, EAR based on 0.3 mg/1000 kcals. Calculated based on energy requirements taken as EARs for 15–18-year-old females (2110 kcals) and 19–50-year-old females (1940 kcals)

^e^Niacin DRVs calculated using LRNI and EAR given as mg NE/1000 kcals (4.4 and 5.5, respectively). Calculated based on energy EAR for 15–18-year-old females (2110 kcals) and 19–50-year-old females (1940 kcals)

^f^Vitamin B6 DRVs calculated using LRNI and EAR in µg/g protein (11 and 13, respectively). Calculated based upon recommendations for 45 g/day of protein and converted into mg

^g^Energy EARs taken from COMA [33] for 15–18-year-old females (2110 kcals) and 19–50-year-old females (1940 kcals)

^h^Ratios are for intake to EAR

### Multiple micronutrient insufficiencies

**Fig. 2:**
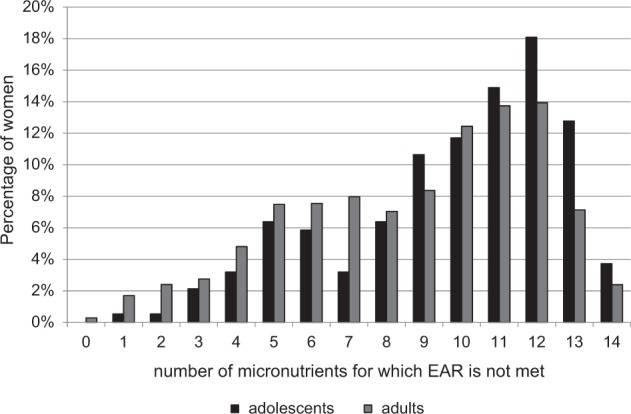
Percentage of women with intakes of multiple micronutrients below the estimated average requirement (EAR)

The majority of women had intakes below the EAR for at least 5 micronutrients, and half for 10 or more (Fig. 2). Half of the adults were not meeting the EAR for 9–14 micronutrients. Half of the adolescents were not meeting the EAR for 10–14 micronutrients. Over half (49.6%) of the adults and 61.2% of adolescents had intakes below the EAR for 10 micronutrients or more (*χ*^2^ = 9.72, *p* < 0.001).

### Important dietary sources of micronutrients

**Fig. 3:**
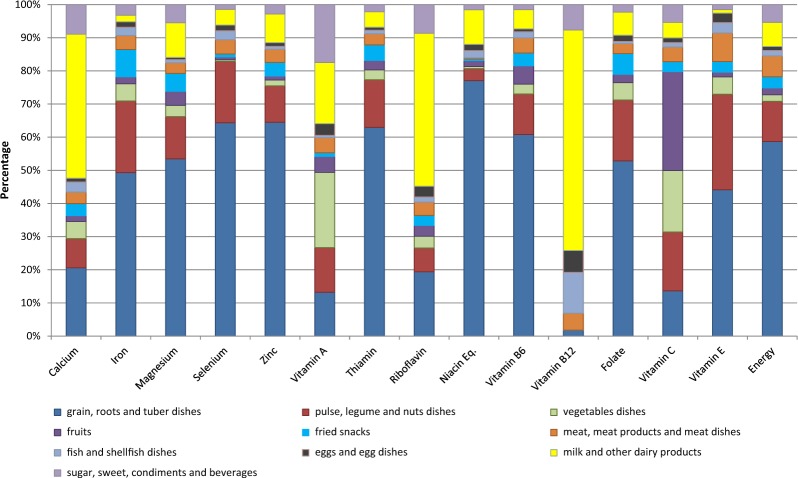
Percentage of micronutrient and energy intakes from 10 food groups

Grains, roots and tubers were important sources of iron, magnesium, selenium, zinc, thiamine, niacin, vitamin B6 and folate (Fig. 3). This group also contributed to vitamin B12 intakes, due to dairy foods, such as ghee, used in these dishes. Pulses, legumes and nuts contributed over 15% of the intakes of iron, selenium, folate and vitamin C.

Despite low intake frequencies, vegetables provided important dietary contributions of vitamin A and vitamin C, and smaller contributions of calcium, iron, thiamine, riboflavin, vitamin B6 and folate. Of the women consuming vegetable dishes, 90% consumed GLVs, which contributed large amounts of the total vitamin A (98%), vitamin C (69%) and other micronutrients (over 70% of each) from vegetable dishes. Fruits contributed around 30% of vitamin C, 6% of vitamin B6 and 5% of vitamin A.

Milk and other dairy products were an important source of calcium (43%), vitamin A (18%), riboflavin (46%) and vitamin B12 (67%). Eggs, fish and shellfish were important sources of vitamin B12, contributing 7% and 12%, respectively, of the total intakes of B12, but contributed <5% of all other micronutrients. Sugar, sweets, condiments and beverages provided <10% of most micronutrients, except vitamin A (17%).

## Discussion

### Micronutrient intakes

We found that women of reproductive age living in Mumbai slums had dietary intakes of many important micronutrients below recommendations, with many having low intakes of multiple micronutrients. Our findings are in line with similar studies in India [10, 11] and other LMICs [2, 8, 16, 39], suggesting that this is a common problem among women of reproductive age. Although low reported intakes are not always associated with clinical deficiency [40–42], studies have demonstrated associations between low intakes and low circulating concentrations of iron [11], zinc [11] and folate [43, 44]. At least 50% of women had intakes of calcium, iron, selenium, vitamin A, riboflavin and niacin below the LRNI, an amount that is sufficient for the 3% of the population with the lowest requirements, indicating a serious risk of deficiency. Thiamine, vitamin B6 and vitamin C were the only nutrients for which at least 50% of women met the EAR, suggesting that some women are not consuming enough of the other micronutrients to meet their needs. Over 50% of women had intakes below the EAR for at least 10 micronutrients concurrently, suggesting that diets are generally nutritionally inadequate. Studies have shown interactions between micronutrients [45–47], indicating that insufficiencies of certain nutrients may exacerbate deficiencies of other micronutrients [48, 49] or reduce the effectiveness of supplementation [43].

### Dietary reference values

The DRVs are intended to indicate the dietary intakes necessary to maintain adequate nutritional status. Each of them was developed using the best available evidence at the time, and we recognise that the robustness of this evidence has changed with time and varies between nutrients, precluding precise conclusions regarding adequacy. For some nutrients, the LRNI, EAR and RNI are inconsistent across the different reference documents [32–35]. This is most marked for selenium; the FAO RNI [32] is substantially lower than the COMA LRNI [33] and American IOM EAR [35]. FAO used the same experimental data as COMA and IOM, but applied different calculations. FAO also assumed lower body weights, appropriate for LMICs. The choice of a boundary to define the reference points within the distribution of requirements, can modify interpretation of the adequacy of the intakes substantially, and since the evidence upon which reference lines are based is limited, there is a continuing need for data to refine them for different populations.

### Strengths and limitations

We collected data from a large, rarely studied population of urban slum-dwelling women before they became pregnant, providing novel information on intakes of several micronutrients. The FFQ featured a comprehensive list of foods derived from extensive community and pilot work. However, FFQs carry recognised potential for imprecision, recall errors and over- and under-reporting. Conversely, the monotonous diets of Indian women may enhance the accuracy. There were no biomarker data with which to validate the FFQ, because there was concern that blood sampling would discourage participation. We used food composition data from a UK-based database, because there was no available up-to-date Indian database. This could introduce error due to differences in nutrient content between UK and Indian foods. For example, selenium content of foods varies significantly according to soil concentrations. Iron bioavailability may have been under-estimated, as iron status has an impact upon absorption, with higher absorption in individuals with lower iron stores [50].

### Adolescents

Adolescent pregnancies carry higher risks of low birth weight and preterm birth, and children of adolescent mothers are at increased risk of stunting and low educational attainment [51]. DRVs for adults and adolescents differ based upon their requirements, with adolescents often requiring more due to ongoing growth. We found that adolescents had significantly lower intakes of magnesium, zinc, thiamine, niacin, vitamin B6 and vitamin E than adult women, and therefore may be at increased risk of deficiencies if they become pregnant.

### Dietary quality

Consistent with other Indian studies, the women had low intakes of micronutrient-rich foods, such as animal source foods, fruit and vegetables [10, 26, 52]. Evidence suggests that poor-quality diets contribute to insufficiencies of multiple micronutrients [32, 53], and adverse maternal and child outcomes [54]. Intakes of carbohydrate-rich foods were high in the sample, which can be common in Indian diets [20], especially in low-income households [16, 55]. This may satisfy hunger and provide sufficient energy, but fails to provide the required quantities of micronutrients (e.g. vitamin B12 [56]) or bioavailable forms (e.g. iron [57]). Meat was consumed infrequently and in small quantities, and therefore contributed minimally to micronutrient intakes in this study. Pulses were important sources of energy, protein, iron, selenium, folate and vitamin C. Recent sharp price rises for pulses have made them unaffordable to poorer consumers and caused further reductions in protein consumption [58].

### Areas for further research

Evidence is lacking on the determinants of poor nutrient intakes in urban slum populations. A study of the determinants would help to inform policy and interventions. There is a lack of evidence on how dietary intakes affect biochemical status, and whether dietary interventions can address deficiencies. International recommendations for improving micronutrient status focus mainly on pregnant and lactating women and young children, due to a strong evidence base of effectiveness [5, 9]. There is growing evidence of the importance of good nutrition in the preconceptional period, but effective, sustainable and scalable interventions are lacking [9, 59, 60].

## Conclusions

Women of reproductive age living in Mumbai slums are consuming diets lacking in a number of important micronutrients, increasing their risk of deficiency, and, in turn, ill-health, adverse birth outcomes and suboptimal child development. There is a need to explore the factors causing poor intakes, in order to direct policy and programme interventions that improve the quality and nutritional sufficiency of diets of low-income women of reproductive age.
